# Association Between Trauma, Impulsivity, and Functioning in Suicide Attempters

**DOI:** 10.3390/bs15091262

**Published:** 2025-09-15

**Authors:** Paula Jhoana Escobedo-Aedo, Alejandro Porras-Segovia, Maria Luisa Barrigón, Philippe Courtet, Jorge López-Castroman, Enrique Baca-Garcia

**Affiliations:** 1 Department of Psychiatry, Hospital Universitario de Torrejón, 28850 Madrid, Spain; pjescobedo@torrejonsalud.com; 2 Department of Psychiatry, Autonomous University of Madrid, 28029 Madrid, Spain; 3 Department of Psychiatry, Hospital Rey Juan Carlos, 28933 Madrid, Spain; alejandro.porras@quironsalud.es; 4 Fundación Jiménez Díaz Health Research Institute (IIS-FJD), 28040 Madrid, Spain; 5 Institute of Psychiatry and Mental Health (IiSGM), Hospital General Universitario Gregorio Marañón, 28007 Madrid, Spain; marialuisa.barrigon@salud.madrid.org; 6 Biomedical Research Networking Center for Mental Health Network (CIBERSAM), 28029 Madrid, Spain; 7 Gregorio Marañón Health Research Institute, 28009 Madrid, Spain; 8 School of Medicine, Universidad Complutense de Madrid, 28040 Madrid, Spain; 9 Department of Emergency Psychiatry and Acute Care, Lapeyronie Hospital, CHU Montpellier, 34090 Montpellier, France; p-courtet@chu-montpellier.fr; 10 Department of Psychiatry, Nimes University Hospital, 30029 Nimes, France; jorge.lopez.castroman@usc.es; 11 CIBERSAM, Research Group CB/07/09/0025, 28007 Madrid, Spain; 12 Department of Psychiatry, Radiology, Public Health, Nursing and Medicine, University of Santiago de Compostela, 15782 Santiago de Compostela, Spain; 13 Department of Psychiatry, Hospital Universitario Fundación Jiménez Díaz, 28040 Madrid, Spain; 14 Department of Psychiatry, Hospital General de Villalba, 28400 Madrid, Spain; 15 Department of Psychiatry, Hospital Universitario Infanta Elena, 28340 Madrid, Spain; 16 Department of Psychology, Universidad Católica del Maule, Talca 3480564, Chile

**Keywords:** trauma, impulsivity, functioning, suicide, suicide attempts, suicide ideation

## Abstract

Suicide is a significant public health concern associated with multiple risk factors. Among these factors, a history of trauma and impulsivity has recently received particular attention. Nevertheless, the relationship between trauma, impulsivity, and functional impairment in individuals who attempt suicide remains to be fully elucidated. This study aimed to examine the association between trauma, impulsivity, and functioning in a clinical sample with previous suicide attempts. A total of 293 patients were included in the study, with a mean age of 41.42 years (SD 14.37 years). The participants had consulted hospitals due to suicide attempts or severe suicidal ideation. The patients were recruited from three hospitals across Spain. Participants completed assessments designed to measure childhood trauma, impulsivity, and functioning. Pearson’s correlations and logistic regression analyses were used to explore associations between trauma, impulsivity, and their impact on functioning. The findings of the present study indicated a modest yet statistically significant correlation between trauma and impulsivity and between impulsivity and functioning. The findings of the logistic regression analysis indicated that physical and sexual abuse, in conjunction with impulsivity, were significant predictors of diminished functioning. The present study found no evidence of a moderating effect of gender or age in the observed relationships. After controlling for all significant variables, impulsivity was the only factor that retained its statistical significance. The present findings underscore the significance of incorporating a focus on impulsivity within clinical interventions targeting individuals who have attempted suicide, with the objective of enhancing their overall functionality.

## 1. Introduction

Suicide is a significant global health issue, with an annual global mortality rate of over 720,000 people. According to the World Health Organization (WHO), suicide is among the top causes of preventable death on a global scale (WHO, 2025).

Research has identified numerous risk factors associated with suicide and suicidal behavior. According to Nock, a history of suicide attempts is a highly significant predictor of future attempts and subsequent death by suicide (Nock, 2009). Suicidal ideation has also been demonstrated to predict suicide attempts (Klonsky et al., 2017). A multitude of additional factors have been identified as being associated with suicidal behavior, including family history of suicidal behavior (Kendler et al., 2023), socioeconomic factors, and substance use (WHO, 2014). Three significant clinical factors have been identified as contributing to suicidal behavior. The aforementioned factors include, but are not limited to, mental disorders, personality traits, and trauma. In the context of psychiatric diagnoses, numerous disorders have been identified as predictors of suicide behavior (see Bertolote et al., 2004; Alvarado-Esquivel et al., 2014). Moreover, clinical research has identified a correlation between personality traits such as aggressiveness and impulsivity (Giner et al., 2014; Lopez-Morinigo et al., 2021; Sanz-Gómez et al., 2024) and emotional dysregulation (Dvir et al., 2014) as well as suicide. A body of research has identified a link between adverse childhood experiences (ACEs) and suicidal behavior (WHO, 2014; M. Liu et al., 2021; Nabinger et al., 2024). It is imperative to consider these factors, as they may be addressed within a clinical context.

The aforementioned factors have been identified as suicide predictors, and associations have been identified among them. For instance, ACEs have been associated with the development of mental disorders (Sahle et al., 2022). Additionally, some authors have proposed that the impact of trauma on suicide risk may be moderated by impulsivity (Dal Santo et al., 2020). This finding aligns with other studies that have identified a link between trauma and impulsivity (Richard-Lepouriel et al., 2019; Johnson, 2025). Consequently, a model could be proposed in which trauma predicts impulsivity, which in turn predicts suicide attempts.

Notwithstanding the identification of these risk factors for suicide, its occurrence remains elevated and highly unpredictable (Turecki et al., 2019). It is imperative that we enhance our comprehension of this global health issue to effectively mitigate its repercussions on the global population.

Conventionally, the global impact of suicide has been evaluated in terms of its impact on mortality, Years of Potential Life Lost (YPLL), and Disability-Adjusted Life Years (DALYs) (WHO, 2018; Naghavi and Global Burden of Disease Self-Harm Collaborators, 2019; GBD 2017 DALYs and HALE Collaborators, 2018). The aforementioned concepts are instrumental in quantifying its repercussions from a global economic perspective. However, it must be noted that the precision of these concepts is far from perfect. The repercussions on the patient’s everyday life bring us closer to the term “functioning.” According to Cambridge, the term “functioning” is defined as “the fact of working or operating, or the way that something operates” or “the normal way that someone’s body or mind works and their ability to do things” (Cambridge Dictionary, 2025). The WHO conceptualizes functioning as the positive or neutral interactions between a specific health condition and contextual factors. These contextual factors have been demonstrated to exert an influence on an individual’s bodily functions and structures, activities, and participation in life situations (WHO, 2001).

Addressing functioning is important because, beyond the diagnosis of a disease, the subsequent repercussions on the patient’s ability to function in various settings, including their home, workplace, educational environment, and other domains, are instrumental in comprehending the clinical picture. This comprehensive approach provides a more nuanced understanding of the disease’s impact on the patient’s life. This assertion is further substantiated by the observation that individuals typically seek medical assistance when they recognize that they are no longer able to function according to their previous standards of wellbeing, rather than due to the presence of a disease (Üstün et al., 2010). According to the scale proposed by the WHO for the assessment of functioning, the areas that may be affected include the following: communication (difficulties in communicating or understanding); mobility (difficulties in moving around); personal care (difficulties in grooming and personal care); relationships (difficulties in relationships with others); activities (difficulties in performing household or work tasks); and participation (difficulties in participating in community activities).

The functionality of an individual may be impacted by any disease, and psychiatric disorders are not an exception to this phenomenon. The present study posits the following research question: How is normal life affected in those attempting suicide? However, the extant evidence on this matter is currently limited. Suicidal ideation and behaviors have been demonstrated to exert an influence on overall functioning (Naghavi and Global Burden of Disease Self-Harm Collaborators, 2019; van Spijker et al., 2020). From another perspective, the presence of low functionality predicts an increased risk of suicide attempts in patients diagnosed with depression and bipolar disorder (Sarhan et al., 2019). In contrast, enhanced functionality has been demonstrated to reduce suicidal ideation (Lutz et al., 2017). This data suggests the presence of an association between functioning and suicide attempts; however, further research is necessary to elucidate the underlying factors contributing to this relationship. It has recently been suggested that exposure to trauma, in addition to its capacity to predict impulsivity, exerts an influence on health-related decision-making processes within specific populations (Meadows, 2025). Furthermore, a relationship has been identified between experiencing trauma and functional impairment across various diagnostic groups (Cotter et al., 2014; Copeland et al., 2018). As postulated by other authors (Bolton et al., 2004), the emergence of psychopathology is related to the occurrence of trauma. The extant data are insufficient for determining the factors that influence alterations in functioning among individuals who have attempted suicide.

The present study aims to contribute to the body of knowledge concerning this issue by analyzing the impact of trauma and impulsivity on functioning. As a secondary outcome, the present study endeavors to analyze the association between trauma and impulsivity. It is hypothesized that, in clinical samples, both trauma and impulsivity have a detrimental effect on patients’ functionality.

## 2. Materials and Methods

### 2.1. Study Setting and Design

This is an observational cross-sectional analysis of baseline data from two studies (SmartCrisis, registration number NCT03720730 and SmartCrisis 2.0, registration number NCT04775160).

The present study was conducted in accordance with the principles delineated in the World Medical Association’s Declaration of Helsinki on Ethical Principles for Medical Research Involving Human Subjects (World Medical Association, 2013). Ethical approval was obtained from the Ethics Committee of the Fundación Jiménez Díaz University Hospital.

The data presented herein was obtained from two prior prospective multicenter studies: SmartCrisis and SmartCrisis 2.0. The following medical centers participated in the aforementioned studies: Fundación Jiménez Díaz University Hospital (Madrid, Spain), Rey Juan Carlos Hospital (Móstoles, Spain), the General Hospital of Villalba (Villalba, Spain), Centre Hospitalier Universitaire Montpellier (Montpellier, France), and the Centre Hospitalier Universitaire Nîmes (Nîmes, France).

The present study utilized a subsample of the aforementioned studies, comprising exclusively Spanish patients, and used a subset of the measures (explained below). The complete protocols for both the SmartCrisis and SmartCrisis 2.0 have been published previously (Berrouiguet et al., 2019; Barrigon et al., 2022).

### 2.2. Sample

The sample population comprised patients who had recently attempted suicide or exhibited severe suicidal ideation. These subjects were recruited from the emergency department or the inpatient or outpatient facilities of any of the aforementioned Spanish hospitals.

The inclusion criteria were as follows:

1. Age of 18 years or older.
2. Attendance at any of the specified clinical services within the past month due to a suicide attempt or emergency referral for suicidal ideation.
3. Ability to understand and sign the informed consent form.
4. Fluency in Spanish (or French in the French centers, though that sample was not utilized in this study), enabling comprehension of the information sheet, informed consent, assessments, and mobile application menus.

Exclusion criteria included the following:

1. Age under 18 years.
2. Inability or unwillingness to provide informed and signed consent.
3. Emergency situations where health status precluded obtaining written consent.

Participants were included regardless of their diagnosis or prior treatment history, including those not receiving any treatment. All patients provided written informed consent. The subjects were informed that their data would be pseudonymized to prevent identification and that the information collected could be used for research purposes. The voluntary nature of participation was emphasized, along with the right to withdraw consent and discontinue participation at any time. No financial compensation or costs were associated with participation in this study.

### 2.3. Measures (Used for This Study)

Sociodemographic variables used for this study were collected during the baseline interview and included the following variables: age, gender, marital status, number of children, and their respective living arrangements.

Clinical suicidal events were determined by a comprehensive review of the patients’ electronic medical records, which included any data referred to as active suicidal ideation and suicide attempts, prior to the baseline interview.

Impulsivity was evaluated using the Barratt Impulsiveness Scale (BIS-11 or BIS), which showed a Cronbach alpha of 0.87 (Martínez-Loredo et al., 2015). This scale has been adapted for the Spanish population (Oquendo et al., 2001). This is a 30-item instrument, rated on a 4-point Likert scale for rating. The assessment encompasses three distinct dimensions: attentional impulsivity (8 items), motor impulsivity (10 items), and non-planning impulsivity (12 items). The concept of self-control is primarily inferred from the non-planning subscale, which is associated with the planning and control of actions, with moderate contributions from the attentional and motor subscales.

ACEs were measured with the Childhood Trauma Questionnaire (CTQ), which has been validated in Spanish with Cronbach’s alpha of 0.89 (Behn et al., 2020) and in some subpopulations (García-Fernández et al., 2024; Hernandez et al., 2013). This self-report instrument comprises 28 items assessing exposure to five types of trauma: emotional, physical, and sexual abuse, as well as emotional and physical neglect.

Functioning was assessed using the WHO Disability Assessment Schedule 2.0 (WHODAS 2.0 or WHODAS). This instrument is characterized by its standardized nature, rendering it applicable across adult populations and cultures. It exhibits an internal consistency (Cronbach’s alpha) of 0.92 (Díaz-Castro et al., 2024). The evaluation encompasses six domains: cognition, mobility, self-care, interpersonal relationships, life activities, and participation.

### 2.4. Procedure

1. Recruitment: The attending psychiatrist or psychologist proposes the patient’s participation in the study. If the patient agrees, they sign the consent form and are included in the study.
2. Baseline Interview: A trained psychologist conducts the initial interview and administers the aforementioned scales.

### 2.5. Data Analysis

Descriptive statistics summarized sociodemographic and clinical variables included in this study. Pearson’s correlation coefficients were calculated to assess the associations between variables such as trauma, impulsivity, and functioning. These analyses were conducted using the Jeffrey’s Amazing Statistics Program (JASP), version 0.19.3.

In order to investigate the association between trauma and impulsivity on functioning, a logistic regression analysis was employed. In this study, age, number of children, and impulsivity were continuous variables, while gender was a dichotomous variable. Marital status was a categorized variable. A disaggregated analysis was conducted for each subitem concerning living situation and trauma.

The dependent variable, functioning, was dichotomized using the 50th percentile of the sample as the cutoff. Patients exhibiting scores above the established cutoff threshold were designated as demonstrating altered functioning, while those falling below the cutoff were classified as exhibiting adequate functioning. Consequently, a regression model was developed, incorporating all statistically significant variables. These analyses were conducted using the Statistical Package for the Social Sciences (SPSS), version 25.0.

Listwise deletion approach was used for missing data. All statistical tests were two-tailed, with a significance level set at 0.05 (*p* < 0.05), and 95% confidence intervals were reported where applicable.

## 3. Results

### 3.1. Sample Characteristics

The present study encompassed a total of 293 patients, of whom 65.5% (*n* = 192) were female. The majority of the sample was single (*n* = 125, 42.8%), followed by those who were married or cohabiting for more than six months (*n* = 101, 34.6%). The data demonstrate that more than half of the patients (*n* = 166, 56.9%) did not have any children, while a smaller percentage had four or more children (*n* = 6, 2.1%). The majority of patients resided with their partner (*n* = 98, 35.1%) or with their family (*n* = 95, 34.1%). Table 1 provides further details regarding the sample characteristics.

### 3.2. Correlation Between Variables

Pearson’s correlation analysis revealed a statistically significant correlation between trauma and impulsivity. Total impulsivity was significantly correlated with all subscales of trauma: emotional abuse (r = 0.362, CI [0.241–0.475], *p* = < 0.001), sexual abuse (r = 0.276, CI [0.138–0.419], *p* = < 0.001), physical abuse (r = 0.329, CI [0.189–0.455], *p* = < 0.001), emotional neglect (r = 0.179, CI [0.043–0.324], *p* = 0.015), and physical neglect (r = 0.314, CI [0.184–0.452], *p* = < 0.001). For a more thorough examination of this correlation and its subscale components, please refer to the Supplementary Material, Table S1 and Figure S1.

Correlations between trauma and functioning differed depending on the type of abuse. The findings of the study showed a statistically significant correlation between physical abuse and all functioning subscales: cognition (r = 0.190, CI [0.064–0.318], *p* = 0.002), mobility (r = 0.176, CI [0.056–0.301], *p* = 0.004), self-care (r = 0.197, CI [0.063–0.352], *p* = 0.001), getting along (r = 0.266, CI [0.131–0.402], *p* < 0.001), life activities (r = 0.160, CI [0.034–0.288], *p* = 0.008), and participation (r = 0.161, CI [0.040–0.282], *p* = 0.011). For a more thorough examination of the correlation between trauma and functioning, please refer to the Supplementary Material, Table S2 and Figure S2.

A subsequent analysis of the Pearson correlation between impulsivity and functioning revealed a significant correlation between total impulsivity and all subscales of functioning: cognition (r = 0.475, CI [0.360–0.573], *p* < 0.001), mobility (r = 0.265, CI [0.142–0.401], *p* < 0.001), self-care (r = 0.254, CI [0.112–0.381], *p* < 0.001), getting along (r = 0.388, CI [0.205–0.535], *p* < 0.001), life activities (r = 0.374, CI [0.229–0.498], *p* < 0.001), and participation (r = 0.375, CI [0.231–0.511], *p* < 0.001). Further details regarding this association can be found in the Supplementary Material, Table S3 and Figure S3.

**Figure 1:**
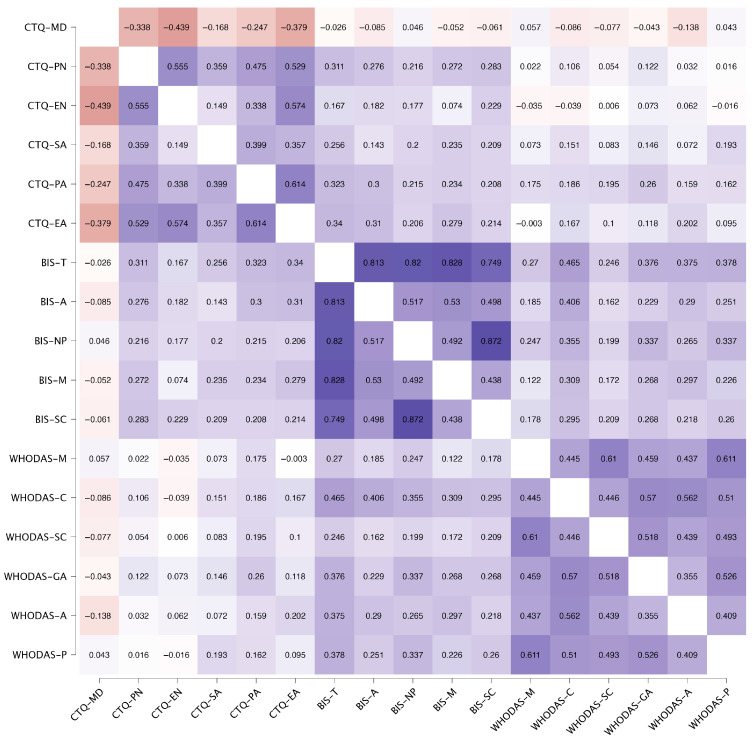
Heatmap of Pearson’s Correlation for trauma, impulsivity, and functioning, adjusted by gender.

Following the adjustment for the confounding variable, gender, the observed associations remained statistically significant, as demonstrated in Figure 1. For a more thorough examination of this analysis, please refer to the Supplementary Material, Table S4.

### 3.3. Factors Associated with Functioning

In the present study, a series of factors were identified as having a significant impact on functioning in the sample population. These factors, which were found to be statistically significant at a *p*-value less than 0.05, included impulsivity (odds ratio [OR] = 1.053, *p* < 0.001), history of sexual abuse (OR = 1.056, *p* = 0.021), and history of physical abuse (OR = 1.058, *p* = 0.021). Further details regarding this analysis are provided in Table 2.

After conducting analysis that incorporated adjustments based on gender and age, the observed association remained statistically significant for impulsivity (OR = 1.051, *p* < 0.001), sexual abuse (OR = 1.051, *p* = 0.039), and physical abuse (OR = 1.062, *p* = 0.015). Further details regarding this analysis can be found in Table 3.

Following the implementation of the multivariable regression model, the sole variable that demonstrated a persistent association with functioning was impulsivity (OR = 1.052, Wald = 10.986, *p* < 0.001). The specifics of this analysis are provided in Table 4.

## 4. Discussion

In this study, we examined the influence of trauma and impulsivity on functioning among people with a history of severe suicidal ideation or suicide attempt. Our findings show that impulsivity is significantly associated with functioning. Also, we found that trauma and impulsivity were significantly correlated.

### 4.1. Demographic Factors

Suicide remains a leading cause of death among adolescents and adults (WHO, 2025). Research has identified gender as a key factor in suicide risk. According to the WHO (2018), males are more likely to die by suicide, while females are more likely to attempt suicide (Miranda-Mendizabal et al., 2019). The observed discrepancies may be ascribed to the influence of socioeconomic and cultural factors. It is noteworthy that research indicates that the roles of women and men, as well as their personality traits, are much more differentiated in developed countries than in less developed countries, where men and women are more alike (Schmitt et al., 2008). In the present sample, which included suicide attempters in Spain, the majority of participants were female, a finding that aligns with the extant scientific evidence. These trends are of interest, but further studies are necessary to fully characterize the factors that shape comparative suicide statistics between men and women.

A significant proportion of the sample was unmarried, with over half of the subjects being childless. These findings deviate from the demographic trends observed in the general populations of Europe and Spain. Recent data indicates that only 19% of women over 45 in Spain remain childless (Adserà & Lozano, 2021), with a mean fertility rate of 1.38 in Europe (Melo, 2025) and 1.12 in Spain (Melo, 2025; Instituto Nacional de Estadística [INE], 2023). These discrepancies may or may not be attributable to the clinical nature of our sample. Furthermore, we could hypothesize that these observed differences are related to altered functioning in our sample. However, the present analysis does not permit the determination of the underlying reasons for this trend. Exploration of these factors in future research would be a valuable avenue for further investigation.

### 4.2. Correlation Between Trauma and Impulsivity

The association between trauma and impulsivity has been the subject of prior research, as evidenced by the work of Braquehais et al. (2010). These researchers posited that ACEs may result in neural modifications in the brains of affected individuals, which could contribute to the development of impulsivity. A subsequent meta-analysis, such as that of R. T. Liu (2019), has demonstrated a robust correlation between childhood trauma and impulsivity, with emotional abuse and sexual abuse exhibiting particularly strong associations (R. T. Liu, 2019). In accordance with the aforementioned assertions, a recent systematic review and meta-analysis corroborated the hypothesis that impulsivity would serve a mediating role in the association between trauma and suicide attempts (Pérez-Balaguer et al., 2022). In accordance with the aforementioned evidence, the present study demonstrated a significant correlation between total impulsivity and all forms of trauma, although this correlation was modest. In the present study, the sole exception was emotional neglect, which demonstrated an absence of a significant correlation with the “motor” impulsivity subscale. This finding suggests that individuals who have experienced a deficiency in care and emotional support from caregivers may exhibit elevated impulsivity scores, devoid of the concomitant participation of altered motor impulsivity. Interestingly, a significant correlation was observed between the attention subscale of impulsivity and all types of trauma (physical, sexual, and emotional abuse as well as physical and emotional neglect). This finding suggests a potential association between trauma and diminished attention, which may explain a response to victimization in certain patients experiencing trauma. This response to trauma may be analogous to the association observed in other clinical disorders, such as attention-deficit/hyperactivity disorder (ADHD). In this disorder, symptoms of inattention have been proposed to be related to trauma, a reason why some authors suggest the need for a correct evaluation of patients before diagnosis (Cornellà Canals & Juárez López, 2014). Also, Richard-Lepouriel et al. (2019) suggested that impulsivity might appear in response to trauma in different disorders (Richard-Lepouriel et al., 2019).

Furthermore, the implications of impulsivity development in relation to traumatic events can vary. For instance, a recent study examining the associations among ACEs, impulsivity, and health-related behaviors found that the impact of ACEs on emotional states can influence impulsivity, potentially giving rise to a pattern of risky health-related attitudes (Meadows, 2025). This finding is of particular value because, in clinical practice, it is common to observe that trauma affects not only psychopathology but also the patient’s engagement in the therapeutic process. This consideration is of the utmost importance in developing clinical approaches for these patients.

While the correlation between trauma and impulsivity appears to be a consistent finding in the extant literature, further research is necessary to elucidate how distinct types of trauma influence specific domains of impulsivity.

### 4.3. Factors Predicting Functioning

The current body of literature suggests a link between trauma and its subsequent impact on adult outcomes (Copeland et al., 2018), with studies indicating functional repercussions in patients with diverse clinical diagnoses (see Cotter et al., 2014; Alameda et al., 2015). This finding aligns with the results of our study, which demonstrated a significant correlation between functioning and trauma, though this correlation varied among different types of trauma. The findings of this study indicate a robust correlation between physical abuse and all subscales of functioning. This finding suggests that individuals who have experienced physical abuse may exhibit impaired performance in multiple domains of functioning. Additionally, a significant correlation was identified between sexual abuse and the cognition and participation subscales of functioning. This finding suggests that this form of abuse may have a more pervasive impact on the victim’s interactions with others. In contrast, emotional neglect demonstrated an absence of correlation with any functioning subscales. Furthermore, the logistic regression analysis revealed a significant association between physical and sexual abuse and functioning, even when adjusting for age and gender. Corroborating these results, extant studies have contributed to delineating particular outcomes associated with distinct forms of trauma. For instance, Moreno-Manso et al. (2022) have indicated that victims of physical abuse and neglect would have a range of repercussions, such as disturbances in attention, organization, and cognition. The authors have identified a stronger association in victims of physical abuse compared to neglect (Moreno-Manso et al., 2022). In this line, Jacob et al. (2020) have shown that both physical and sexual abuse during childhood are associated with altered adult functioning (Jacob et al., 2020). These effects would not only be observed in adulthood but would also be evident from school age onward. As Mitchell et al. (2021) indicate, victims of sexual abuse would have a lower likelihood of continuing schooling or attending college (Mitchell et al., 2021).

Similarly, in our sample, sexual and physical abuse were the only types of abuse that showed a statistically significant association with functioning. While this evidence provides important information and begins to delineate possible patterns related to traumatic experiences, it is important that future studies provide more information to corroborate or better define this association.

Additionally, a statistically significant correlation was identified between impulsivity and functioning subscales, with the exception of motor impulsivity. It is noteworthy that motor impulsivity did not demonstrate a significant association with the “mobility” functioning subscale, suggesting that individuals with high motor impulsivity scores may experience fewer mobility limitations. Consequently, the present findings suggest that impulsivity exerts a significant influence on multiple domains of patient functioning, thereby underscoring its substantial clinical implications.

Preliminary research has indicated the presence of alterations in specific cognitive functions associated with impulsivity (Kockler & Stanford, 2008). With respect to the consequences of impulsivity on functioning, existing studies have primarily concentrated on specific diagnoses, such as bipolar disorder, where impulsivity has been associated with impaired quality of life (Jiménez et al., 2012), and ADHD, where the failure to address impulsivity symptoms in individuals diagnosed with ADHD has been observed to adversely impact their overall functioning (Papazian et al., 2002). However, these studies have not been extensive enough to encompass the broader clinical population.

Furthermore, in our sample, after controlling for sex and age, and subsequently performing the multivariate analysis, impulsivity was the only variable that remained significant in its association with functioning. Therefore, the findings of this study suggest that elevated impulsivity levels are associated with suboptimal functioning in individuals who have attempted suicide. No statistically significant associations were identified with regard to gender or age. In contrast, the impact of trauma on functioning lost significance following multivariate analysis. This result suggests a potential association between trauma and functioning, which may be mediated by impulsivity.

It is imperative to acknowledge that the sample population under investigation consists of patients who exhibited severe suicidal ideation or have attempted suicide. This observation suggests the possibility that impulsivity may be particularly pronounced in the present sample. Some authors have noted the association between impulsivity and suicide attempts (Baca-Garcia et al., 2005), although this association has been contested (Anestis et al., 2014). Contrary to this, recent studies have indicated that impulsivity could mediate the association between trauma and suicide attempts (Pérez-Balaguer et al., 2022), and that impulsivity is one of the significant factors in patients with multiple suicide attempts (repeaters) in adult patients (Abascal-Peiró et al., 2023). However, this association has not been observed in the adolescent population (Ezquerra et al., 2023).

The findings of the present study suggest that trauma acts as a possible moderator of impulsivity, which in turn predicts impaired functioning. This finding helps to address the limited body of evidence regarding factors that predict functioning.

### 4.4. Limitations

We acknowledge several limitations of our study. First, the sample was drawn from a clinical population of individuals who had attempted suicide, which may limit the generalizability of the findings to broader populations. Secondly, the correlation between suicide attempts and impulsivity may introduce a bias in the association observed in the present study’s results. Thirdly, the recruitment process primarily occurred in emergency department settings following suicide attempts, which may have resulted in the amplification of the association between certain variables due to the nature of the context. Finally, we acknowledge the limitations of the utilized data, which is characterized by a paucity of variables pertaining to clinical diagnosis and pharmacological treatment. This limitation renders us unable to study all possible contributing variables in this association.

Nevertheless, these findings offer novel insights that may be relevant for the knowledge of suicidal behaviors and the development of targeted therapeutic strategies.

## 5. Conclusions

The present study identified significant associations between the variables examined in a clinical sample of suicide attempters. A statistically significant correlation was observed between the presence of trauma and impulsivity. Finally, impulsivity demonstrated an inverse correlation with patient functioning, indicating that higher levels of impulsivity were associated with lower levels of functioning. Following the implementation of a logistic regression analysis, a significant association was identified between impaired functioning and physical and sexual abuse, as well as impulsivity. Nonetheless, following a multivariate analysis, impulsivity emerged as the sole variable that demonstrated a significant association with functioning.

These findings, though modest, offer valuable insights that may be corroborated in future studies. It is recommended that future research enhances our understanding of functional impairment among individuals who attempt suicide as well as the relation between contributing variables.

## Figures and Tables

**Table 1:** Characteristics of the sample (*n* = 293).

		*n*	%	Mean (SD)	Missing
Age		292		41.42 (14.37)	1
Gender	Male	101	34.47		
	Female	192	65.53		
Marital Status	Single	125	42.81		1
	Married/living with partner > 6 months	101	34.59		
	Divorced	59	20.21		
	Widowed	7	2.40		
Lives with	Couple	98	35.13		14
	Family	95	34.05		
	Alone	58	20.79		
	Institution	1	0.36		
Have children?	None	166	56.85		1
	1	53	18.51		
	2	51	17.47		
	3	16	5.48		
	4 or more	6	2.05		
Impulsivity	Attentional	221		20.15 (4.51)	72
	Motor	201		24.15 (5.57)	92
	Non-planning	211		26.00 (5.47)	82
Trauma	Emotional Abuse	289		13.38 (6.27)	4
	Physical Abuse	284		8.89 (5.46)	9
	Sexual Abuse	278		8.91 (5.70)	15
	Emotional Neglect	292		13.50 (5.20)	1
	Physical Neglect	290		8.68 (4.21)	3
Functioning	Cognition	276		29.67 (22.01)	17
	Mobility	277		21.10 (23.98)	16
	Self-care	283		17.17 (22.00)	10
	Getting along	196		34.23 (29.63)	97
	Life Activities	281		37.92 (30.54)	12
	Participation	255		41.95 (20.42)	38

Abbreviations: SD = standard deviation.

**Table 2:** Factors associated with functioning. Results of the logistic regressions.

Variables		d*f*	OR	95% CI	*p*
Age		1	0.990	0.974–1.007	0.254
Gender	Male		1.00		
	Female	1	1.229	0.759–1.990	0.402
Marital Status		3			0.681
	Married/living with partner > 6 months		1.00		
	Single	1	1.156	0.671–1.991	0.602
	Divorced	1	1.202	0.611–2.367	0.593
	Widowed	1	0.488	0.103–2.297	0.364
Lives with	Couple	1	1.144	0.683–1.915	0.609
	Family	1	1.196	0.712–2.007	0.499
	Alone		1.385	0.744–2.577	0.304
Have children?		1	0.955	0.787–1.159	0.639
**Impulsivity (Barrat Total)**		**1**	**1.053**	**1.025–1.081**	**<0.001**
Trauma	Emotional Neglect	1	0.996	0.951–1.042	0.852
	Physical Neglect	1	0.990	0.935–1.048	0.742
	**Sexual Abuse**	**1**	**1.056**	**1.008–1.106**	**0.021**
	**Physical Abuse**	**1**	**1.058**	**1.009–1.110**	**0.021**
	Emotional Abuse	1	1.021	0.982–1.061	0.289
	Minimization/denial	1	1.051	0.968–1.141	0.234

Abbreviations: CI = confidence interval; d*f* = degrees of freedom; OR = odds ratio.

**Table 3:** Factors associated with functioning, adjusted by age and sex.

Variables		OR	95% CI	*p*
Trauma	Emotional neglect	0.991	0.946–1.038	0.713
	Emotional Abuse	1.016	0.977–1057	0.425
	**Sexual abuse**	**1.051**	**1.** **003–1.103**	**0.** **039**
	**Physical abuse**	**1.062**	**1** **.012–1.114**	**0.** **015**
**Impulsivity (Total Barrat)**		**1** **.051**	**1.** **023–1.080**	**<** **0.** **001**

Abbreviations: CI = confidence interval; OR = odds Ratio.

**Table 4:** Factors associated with functioning. Multivariable regression model.

Model	B	SE	Wald	d*f*	OR	CI	*p*
Gender	−0.008	0.347	0.001	1	0.992	0.502–1.959	0.981
Age	−0.014	0.012	1.304	1	0.986	0.962–1.010	0.254
Barrat Total	0.050	0.015	10.986	1	1.052	1.021–1.083	<0.001
Physical abuse	0.032	0.038	0.728	1	1.033	0.959–1.111	0.393
Sexual Abuse	0.027	0.037	0.510	1	0.974	0.905–1.047	0.475

Abbreviations: CI = confidence interval; d*f* = degrees of freedom; OR = odds Ratio; SE = standard error.

## Data Availability

The datasets generated during and/or analyzed during the current study are available from the corresponding author on reasonable request.

## Supplementary Materials

The following supporting information can be downloaded at: https://www.mdpi.com/article/10.3390/bs15091262/s1, Table S1. Pearson’s partial correlation between trauma and impulsivity; Table S2. Pearson’s partial correlation between trauma and functioning; Table S3. Pearson’s partial correlation between functioning and impulsivity; Table S4. Pearson’s partial correlation between trauma, impulsivity, and functioning; Figure S1. Heat map of Pearson’s correlation between trauma and impulsivity; Figure S2. Heat map of Pearson’s correlation between trauma and functioning; Figure S3. Heat map of Pearson’s correlation between functioning and impulsivity.
