# Supplementary material for: Association Between Trauma, Impulsivity, and Functioning in Suicide Attempters
Source: Behav Sci (Basel). 2025 Sep 15;15(9):1262. doi: 10.3390/bs15091262 (PMC12466635; doi:10.3390/bs15091262)

Supplementary Material.

Table S1. Pearson's partial correlation between trauma and impulsivity

Variable		CTQ-MD	CTQ-PN	CTQ-EN	CTQ-SA	CTQ-PA	CTQ-EA	BIS-T	BIS-NP	BIS-A	BIS-SC	BIS-M
1.CTQ-MD	(r)	—										
	p-value	—										
		—										
		—										
2. CTQ-PN	(r)	**-0.339**	—									
	p-value	**< 0.001**	—									
	Lower 95% CI	-0.450	—									
	Upper 95% CI	-0.222	—									
3. CTQ-EN	(r)	**-0.442**	**0.556**	—								
	p-value	**< 0.001**	**< 0.001**	—								
	Lower 95% CI	-0.536	0.472	—								
	Upper 95% CI	-0.347	0.626	—								
4. CTQ-SA	(r)	**-0.179**	**0.359**	**0.161**	—							
	p-value	**0.003**	**< 0.001**	**0.007**	—							
	Lower 95% CI	-0.298	0.251	0.037	—							
	Upper 95% CI	-0.060	0.470	0.274	—							
5. CTQ-PA	(r)	**-0.248**	**0.476**	**0.340**	**0.398**	—						
	p-value	**< 0.001**	**< 0.001**	**< 0.001**	**< 0.001**	—						
	Lower 95% CI	-0.354	0.360	0.235	0.268	—						
	Upper 95% CI	-0.1.43	0.578	0.435	0.524	—						
6. CTQ-EA	(r)	**-0.384**	**0.522**	**0.573**	**0.389**	**0.606**	—					
	p-value	**< 0.001**	**< 0.001**	**< 0.001**	**< 0.001**	**< 0.001**	—					
	Lower 95% CI	-0.480	0.439	0.490	0.294	0.530	—					
	Upper 95% CI	-0.286	0.596	0.644	0.486	0.676	—					
7. BIS-T	(r)	-0.038	**0.314**	**0.179**	**0.276**	**0.329**	**0.362**	—				
	p-value	0.609	**< 0.001**	**0.015**	**< 0.001**	**< 0.001**	**< 0.001**	—				
	Lower 95% CI	-0.177	0.184	0.043	0.138	0.189	0.241	—				
	Upper 95% CI	0.106	0.452	0.324	0.419	0.455	0.475	—				
8. BIS-NP	(r)	0.040	**0.219**	**0.184**	**0.213**	**0.221**	**0.221**	**0.823**	—			
	p-value	0.567	**0.001**	**0.007**	**0.002**	**0.001**	**0.001**	**< 0.001**	—			
	Lower 95% CI	-0.097	0.096	0.037	0.084	0.079	0.086	0.764	—			
	Upper 95% CI	0.174	0.354	0.320	0.347	0.348	0.339	0.861	—			
9. BIS-A	(r)	-0.089	**0.278**	**0.188**	**0.157**	**0.303**	**0.320**	**0.816**	**0.522**	—		
	p-value	0.187	**<0.001**	**0.005**	**0.021**	**< 0.001**	**< 0.001**	**< 0.001**	**< 0.001**	—		
	Lower 95% CI	-0.206	0.161	0.066	0.026	0.177	0.205	0.763	0.409	—		
	Upper 95% CI	0.030	0.395	0.310	0.278	0.421	0.431	0.863	0.624	—		
10. BIS-SC	(r)	-0.069	**0.286**	**0.237**	**0.225**	**0.213**	**0.231**	**0.753**	**0.873**	**0.503**	—	
	p-value	0.297	**< 0.001**	**< 0.001**	**< 0.001**	**0.001**	**< 0.001**	**< 0.001**	**< 0.001**	**< 0.001**	—	
	Lower 95% CI	-0.205	0.154	0.106	0.108	0.085	0.105	0.679	0.828	0.390	—	
	Upper 95% CI	0.058	0.414	0.360	0.341	0.336	0.349	0.813	0.908	0.609	—	
11. BIS-M	(r)	-0.061	**0.274**	0.083	**0.252**	**0.239**	**0.298**	**0.831**	**0.498**	**0.536**	**0.444**	—
	p-value	0.394	**< 0.001**	0.242	**< 0.001**	**< 0.001**	**< 0.001**	**< 0.001**	**< 0.001**	**< 0.001**	**< 0.001**	—
	Lower 95% CI	-0.194	0.146	-0.044	0.109	0.100	0.167	0.782	0.377	0.435	0.314	—
	Upper 95% CI	0.068	0.400	0.215	0.395	0.371	0.416	0.871	0.592	0.628	0.556	—

Abbreviations: CTQ=Childhood Trauma Questionnaire, MD=Minimization/Denial, PN=Physical Neglect, EN=Emotional Neglect, SA=Sexual Abuse, PA=Physical Abuse, EA=Emotional Abuse, BIS=Barratt Impulsiveness Scale, T=Total, NP=Non-Planning, A=Attention, SC=Self-Control, M=Motor.

Supplementary Table S2. Pearson's partial correlation between trauma and functionality

Variable		CTQ-MD	CTQ-PN	CTQ-EN	CTQ-SA	CTQ-PA	CTQ-EA	WHODAS-P	WHODAS-A	WHODAS-GA	WHODAS-SC	WHODAS-M	WHODAS-C
1. CTQ-MD	(r)	—											
	p-value	—											
	Lower 95% CI	—											
	Upper 95% CI	—											
2. CTQ-PN	(r)	**-0.339**	—										
	p-value	**< 0.001**	—										
	Lower 95% CI	-0.446	—										
	Upper 95% CI	-0.230	—										
3. CTQ-EN	(r)	**-0.442**	**0.556**	—									
	p-value	**< 0.001**	**< 0.001**	—									
	Lower 95% CI	-0.534	0.475	—									
	Upper 95% CI	-0.352	0.623	—									
4. CTQ-SA	(r)	**-0.179**	**0.359**	**0.161**	—								
	p-value	**0.003**	**< 0.001**	**0.007**	—								
	Lower 95% CI	-0.299	0.251	0.037	—								
	Upper 95% CI	-0.060	0.460	0.276	—								
5. CTQ-PA	(r)	**-0.248**	**0.476**	**0.340**	**0.398**	—							
	p-value	**< 0.001**	**< 0.001**	**< 0.001**	**< 0.001**	—							
	Lower 95% CI	-0.357	0.359	0.236	0.276	—							
	Upper 95% CI	-0.138	0.571	0.434	0.518	—							
6. CTQ-EA	(r)	**-0.384**	**0.522**	**0.573**	**0.389**	**0.606**	—						
	p-value	**< 0.001**	**< 0.001**	**< 0.001**	**< 0.001**	**< 0.001**	—						
	Lower 95% CI	-0.477	0.440	0.491	0.293	0.529	—						
	Upper 95% CI	-0.281	0.595	0.651	0.475	0.668	—						
7. WHODAS-P	(r)	0.040	0.016	-0.013	**0.209**	**0.161**	0.108	—					
	p-value	0.528	0.804	0.834	**0.001**	**0.011**	0.087	—					
	Lower 95% CI	-0.087	-0.116	-0.131	0.092	0.040	-0.004	—					
	Upper 95% CI	0.165	0.148	0.110	0.325	0.282	0.226	—					
8. WHODAS-A	(r)	**-0.142**	0.034	0.067	0.085	**0.160**	**0.212**	**0.411**	—				
	p-value	**0.019**	0.575	0.263	0.166	**0.008**	**< 0.001**	**< 0.001**	—				
	Lower 95% CI	-0.256	-0.096	-0.058	-0.032	0.034	0.089	0.301	—				
	Upper 95% CI	-0.021	0.165	0.175	0.217	0.288	0.317	0.514	—				
9.WHODAS-GA	(r)	-0.046	0.118	0.080	**0.167**	**0.266**	**0.146**	**0.531**	**0.364**	—			
	p-value	0.527	0.102	0.263	**0.022**	**< 0.001**	**0.043**	**< 0.001**	**< 0.001**	—			
	Lower 95% CI	-0.192	-0.033	-0.063	0.043	0.131	0.007	0.379	0.211	—			
	Upper 95% CI	0.103	0.269	0.231	0.306	0.402	0.283	0.649	0.490	—			
10. WHODAS-SC	(r)	-0.082	0.057	0.018	0.112	**0.197**	**0.128**	**0.498**	**0.443**	**0.530**	—		
	p-value	0.175	0.343	0.768	0.066	**0.001**	**0.033**	**< 0.001**	**< 0.001**	**< 0.001**	—		
	Lower 95% CI	-0.195	-0.074	-0.095	-0.015	0.063	0.011	0.356	0.340	0.406	—		
	Upper 95% CI	0.034	0.187	0.120	0.246	0.352	0.228	0.610	0.541	0.643	—		
11. WHODAS-M	(r)	0.054	0.024	-0.029	0.086	**0.176**	0.013	**0.612**	**0.440**	**0.468**	**0.612**	—	
	p-value	0.380	0.689	0.632	0.162	**0.004**	0.830	**< 0.001**	**< 0.001**	**< 0.001**	**< 0.001**	—	
	Lower 95% CI	-0.068	-0.093	-0.146	-0.039	0.056	-0.102	0.509	0.336	0.355	0.530	—	
	Upper 95% CI	0.172	0.148	0.087	0.210	0.301	0.128	0.692	0.543	0.576	0.690	—	
12. WHODAS-C	(r)	-0.094	0.108	-0.029	**0.183**	**0.190**	**0.194**	**0.515**	**0.566**	**0.579**	**0.457**	**0.449**	—
	p-value	0.123	0.073	0.634	**0.003**	**0.002**	**0.001**	**< 0.001**	**< 0.001**	**< 0.001**	**< 0.001**	**< 0.001**	—
	Lower 95% CI	-0.226	-0.023	-0.149	0.071	0.064	0.069	0.380	0.466	0.470	0.351	0.342	—
	Upper 95% CI	0.036	0.251	0.095	0.302	0.318	0.309	0.619	0.658	0.672	0.563	0.548	—

Abbreviations: CTQ=Childhood Trauma Questionnaire, MD=Minimization/Denial, PN=Physical Neglect, EN=Emotional Neglect, SA=Sexual Abuse, PA=Physical Abuse, EA=Emotional Abuse, WHODAS=World Health Organization Disability Assessment Schedule, P=Participation, A=Activities, GA=Getting Along, SC=Self-Care, M=Mobility, C=Cognition.

Supplementary Table S3. Pearson's partial correlation between functionality and impulsivity

Variable		WHODAS-P	WHODAS-A	WHODAS-GA	WHODAS-SC	WHODAS-M	WHODAS-C	BIS-T	BIS-A	BIS-NP	BIS-SC	BIS-M
1.WHODAS-P	(r)	—										
	p-value	—										
	Lower 95% CI	—										
	Upper 95% CI	—										
2.WHODAS-A	(r)	**0.411**	—									
	p-value	**<0.001**	—									
	Lower 95% CI	0.287										
	Upper 95% CI	0.521										
3.WHODAS-GA	(r)	**0.531**	**0.364**	—								
	p-value	**<0.001**	**<0.001**	—								
	Lower 95% CI	0.381	0.216	—								
	Upper 95% CI	0.656	0.502	—								
4. WHODAS-SC	(r)	**0.498**	**0.443**	**0.530**	—							
	p-value	**<0.001**	**<0.001**	**<0.001**	—							
	Lower 95% CI	0.344	0.339	0.405	—							
	Upper 95% CI	0.610	0.535	0.642	—							
5. WHODAS-M	(r)	**0.612**	**0.440**	**0.468**	**0.612**	—						
	p-value	**<0.001**	**<0.001**	**<0.001**	**< 0.001**	—						
	Lower 95% CI	0.502	0.326	0.345	0.527	—						
	Upper 95% CI	0.697	0.538	0.582	0.685	—						
6. WHODAS-C	(r)	**0.515**	**0.566**	**0.579**	**0.457**	**0.449**	—					
	p-value	**<0.001**	**<0.001**	**<0.001**	**<0.001**	**<0.001**	—					
	Lower 95% CI	0.384	0.461	0.467	0.344	0.335	—					
	Upper 95% CI	0.634	0.652	0.670	0.563	0.548	—					
7. BIS-T	(r)	**0.375**	**0.374**	**0.388**	**0.254**	**0.265**	**0.475**	—				
	p-value	**<0.001**	**<0.001**	**<0.001**	**<0.001**	**<0.001**	**<0.001**	—				
	Lower 95% CI	0.231	0.229	0.205	0.112	0.142	0.360	—				
	Upper 95% CI	0.511	0.498	0.535	0.381	0.401	0.573	—				
8. BIS-A	(r)	**0.254**	**0.291**	**0.239**	**0.168**	**0.185**	**0.413**	**0.816**	—			
	p-value	**<0.001**	**<0.001**	**0.004**	**0.014**	**0.007**	**<0.001**	**<0.001**	—			
	Lower 95% CI	0.119	0.163	0.065	0.046	0.056	0.292	0.764	—			
	Upper 95% CI	0.392	0.415	0.401	0.279	0.320	0.518	0.861	—			
9. BIS-NP	(r)	**0.339**	**0.267**	**0.349**	**0.207**	**0.248**	**0.363**	**0.823**	**0.522**	—		
	p-value	**< 0.001**	**<0.001**	**<0.001**	**0.003**	**<0.001**	**<0.001**	**<0.001**	**<0.001**	—		
	Lower 95% CI	0.216	0.119	0.168	0.058	0.124	0.237	0.768	0.406	—		
	Upper 95% CI	0.454	0.397	0.507	0.346	0.367	0.478	0.864	0.630	—		
10. BIS-SC	(r)	**0.265**	**0.223**	**0.283**	**0.222**	**0.182**	**0.307**	**0.753**	**0.503**	**0.873**	—	
	p-value	**<0.001**	**<0.001**	**<0.001**	**<0.001**	**0.007**	**<0.001**	**<0.001**	**<0.001**	**< 0.001**	—	
	Lower 95% CI	0.130	0.078	0.113	0.076	0.056	0.181	0.683	0.394	0.823	—	
	Upper 95% CI	0.392	0.355	0.432	0.341	0.311	0.419	0.814	0.609	0.906	—	
11. BIS-M	(r)	**0.225**	**0.300**	**0.277**	**0.180**	0.122	**0.319**	**0.831**	**0.536**	**0.498**	**0.444**	—
	p-value	**0.003**	**<0.001**	**0.001**	**0.012**	0.093	**<0.001**	**<0.001**	**<0.001**	**< 0.001**	**< 0.001**	—
	Lower 95% CI	0.069	0.174	0.115	0.051	-0.011	0.190	0.781	0.430	0.387	0.325	—
	Upper 95% CI	0.364	0.435	0.410	0.303	0.274	0.443	0.873	0.635	0596	0.562	—

Abbreviations: WHODAS=World Health Organization Disability Assessment Schedule, P=Participation, A=Activities, GA=Getting Along, SC=Self-Care, M=Mobility, C=Cognition, BIS=Barratt Impulsiveness Scale, T=Total, NP=Non-Planning, A=Attention, SC=Self-Control, M=Motor

Supplementary Table S4. Pearson's partial correlation between trauma, impulsivity and functionality, adjusted for gender.

Variable		CTQ-DM	CTQ-PN	CTQ-EN	CTQ-SA	CTQ-PA	CTQ-EA	BIS-T	BIS-A	BIS-NP	BIS-M	BIS-SC	WHODAS-M	WHODAS-C	WHODAS-SC	WHODAS-GA	WHODAS-A	WHODAS-P
CTQ-DM	(r)	—																
	p-value	—																
	Lower 95% CI	—																
	Upper 95% CI	—																
CTQ-PN	(r)	**-0.338**	—															
	p-value	**<0.001**	—															
	Lower 95% CI	-0.443	—															
	Upper 95% CI	-0.227	—															
CTQ-EN	(r)	**-0.439**	**0.555**	—														
	p-value	**<0.001**	**<0.001**	—														
	Lower 95% CI	-0.538	0.478	—														
	Upper 95% CI	-0.339	0.630	—														
CTQ-SA	(r)	**-0.168**	**0.359**	**0.149**	—													
	p-value	**0.005**	**<0.001**	**0.013**	—													
	Lower 95% CI	-0.278	0.253	0.027	—													
	Upper 95% CI	-0.070	0.471	0.268	—													
CTQ-PA	(r)	**-0.247**	**0.475**	**0.338**	**0.399**	—												
	p-value	**<0.001**	**<0.001**	**<0.001**	**<0.001**	—												
	Lower 95% CI	-0.356	0.364	0.235	0.262	—												
	Upper 95% CI	-0.137	0.580	0.432	0.519	—												
CTQ-EA	(r)	**-0.379**	**0.529**	**0.574**	**0.357**	**0.614**	—											
	p-value	**<0.001**	**<0.001**	**<0.001**	**<0.001**	**<0.001**	—											
	Lower 95% CI	-0.471	0.453	0.493	0.249	0.543	—											
	Upper 95% CI	-0.271	0.605	0.651	0.457	0.675	—											
BIS-T	(r)	-0.026	**0.311**	**0.167**	**0.256**	**0.323**	**0.340**	—										
	p-value	0.729	**<0.001**	**0.024**	**<0.001**	**<0.001**	**<0.001**	—										
	Lower 95% CI	-0.168	0.179	0.030	0.119	0.192	0.210	—										
	Upper 95% CI	0.115	0.438	0.298	0.398	0.448	0.458	—										
BIS-A	(r)	-0.085	**0.276**	**0.182**	**0.143**	**0.300**	**0.310**	**0.813**	—									
	p-value	0.208	**< 0.001**	**0.007**	**0.036**	**< 0.001**	**< 0.001**	**<0.001**	—									
	Lower 95% CI	-0.223	0.151	0.062	0.015	0.179	0.190	0.752	—									
	Upper 95% CI	0.049	0.389	0.310	0.271	0.412	0.428	0.869	—									
BIS-NP	(r)	0.046	**0.216**	**0.177**	**0.200**	**0.215**	**0.206**	**0.820**	**0.517**	—								
	p-value	0.513	**0.002**	**0.010**	**0.004**	**0.002**	**0.003**	**<0.001**	**<0.001**	—								
	Lower 95% CI	-0.075	0.087	0.033	0.081	0.082	0.066	0.765	0.395	—								
	Upper 95% CI	0.186	0.350	0.315	0.343	0.349	0.335	0.860	0.619	—								
BIS-M	(r)	-0.052	**0.272**	0.074	**0.235**	**0.234**	**0.279**	**0.828**	**0.530**	**0.492**	—							
	p-value	0.468	**<0.001**	0.299	**<0.001**	**<0.001**	**<0.001**	**<0.001**	**<0.001**	**<0.001**	—							
	Lower 95% CI	-0.177	0.131	-0.050	0.086	0.093	0.146	0.779	0.421	0.381	—							
	Upper 95% CI	0.081	0.398	0.210	0.377	0.356	0.400	0.872	0.627	0.595	—							
BIS-SC	(r)	-0.061	**0.283**	**0.229**	**0.209**	**0.208**	**0.214**	**0.749**	**0.498**	**0.872**	**0.438**	—						
	p-value	0.352	**<0.001**	**<0.001**	**0.002**	**0.002**	**0.001**	**<0.001**	**<0.001**	**<0.001**	**<0.001**	—						
	Lower 95% CI	-0.185	0.157	0.094	0.093	0.081	0.084	0.675	0.374	0.830	0.309	—						
	Upper 95% CI	0.072	0.405	0.359	0.336	0.332	0.334	0.811	0.600	0.907	0.556	—						
WHODAS-M	(r)	0.057	0.022	-0.035	0.073	**0.175**	-0.003	**0.270**	**0.185**	**0.247**	0.122	**0.178**	—					
	p-value	0.353	0.714	0.566	0.240	**0.004**	0.961	**<0.001**	**0.008**	**<0.001**	0.093	**0.008**	—					
	Lower 95% CI	-0.064	-0.103	-0.149	-0.050	0.051	-0.123	0.152	0.065	0.125	-0.010	0.054	—					
	Upper 95% CI	0.184	0.151	0.092	0.205	0.296	0.121	0.398	0.320	0.362	0.262	0.310	—					
WHODAS-C	(r)	-0.086	0.106	-0.039	**0.151**	**0.186**	**0.167**	**0.465**	**0.406**	**0.355**	**0.309**	**0.295**	**0.445**	—				
	p-value	0.160	0.081	0.516	**0.014**	**0.002**	**0.006**	**<0.001**	**<0.001**	**<0.001**	**<0.001**	**<0.001**	**<0.001**	—				
	Lower 95% CI	-0.209	-0.023	-0.162	0.024	0.052	0.042	0.351	0.284	0.238	0.172	0.176	0.327	—				
	Upper 95% CI	0.043	0.241	0.088	0.270	0.309	0.283	0.569	0.519	0.468	0.425	0.414	0.545	—				

WHODAS-SC	(r)	-0.077	0.054	0.006	0.083	**0.195**	0.100	**0.246**	**0.162**	**0.199**	**0.172**	**0.209**	**0.610**	**0.446**	—			
	p-value	0.204	0.365	0.924	0.176	**0.001**	0.096	**0.001**	**0.018**	**0.005**	**0.017**	**0.002**	**<0.001**	**<0.001**	—			
	Lower 95% CI	-0.190	-0.076	-0.101	-0.049	0.059	-0.019	0.100	0.045	0.057	0.041	0.081	0.529	0.327	—			
	Upper 95% CI	0.047	0.186	0.116	0.208	0.328	0.220	0.377	0.271	0.337	0.297	0.337	0.689	0.550	—			
WHODAS-GA	(r)	-0.043	0.122	0.073	**0.146**	**0.260**	0.118	**0.376**	**0.229**	**0.337**	**0.268**	**0.268**	**0.459**	**0.570**	**0.518**	—		
	p-value	0.557	0.091	0.314	**0.045**	**<0.001**	0.102	**<0.001**	**0.005**	**<0.001**	**0.002**	**<0.001**	**<0.001**	**<0.001**	**<0.001**	—		
	Lower 95% CI	-0.188	-0.025	-0.064	0.013	0.115	-0.016	0.200	0.070	0.164	0.105	0.106	0.350	0.453	0.387	—		
	Upper 95% CI	0.108	0.277	0.215	0.272	0.387	0.253	0.538	0.380	0.513	0.417	0.426	0.574	0.665	0.629	—		
WHODAS-A	(r)	**-0.138**	0.032	0.062	0.072	**0.159**	**0.202**	**0.375**	**0.290**	**0.265**	**0.297**	**0.218**	**0.437**	**0.562**	**0.439**	**0.355**	—	
	p-value	**0.023**	0.599	0.302	0.243	**0.009**	**<0.001**	**<0.001**	**<0.001**	**<0.001**	**<0.001**	**0.001**	**<0.001**	**<0.001**	**<0.001**	**<0.001**	—	
	Lower 95% CI	-0.255	-0.091	-0.058	-0.063	0.030	0.081	0.244	0.168	0.128	0.158	0.084	0.322	0.463	0.332	0.197	—	
	Upper 95% CI	-0.017	0.159	0.184	0.208	0.282	0.311	0.497	0.405	0.398	0.423	0.351	0.539	0.657	0.536	0.498	—	
WHODAS-P	(r)	0.043	0.016	-0.016	**0.193**	**0.162**	0.095	**0.378**	**0.212**	**0.337**	**0.226**	**0.260**	**0.611**	**0.510**	**0.493**	**0.526**	**0.409**	—
	p-value	0.496	0.800	0.795	**0.003**	**0.010**	0.135	**<0.001**	**0.003**	**<0.001**	**0.003**	**<0.001**	**<0.001**	**<0.001**	**<0.001**	**<0.001**	**<0.001**	—
	Lower 95% CI	-0.097	-0.116	-0.143	0.078	0.035	-0.025	0.239	0.125	0.213	0.086	0.130	0.500	0.384	0.347	0.381	0.293	—
	Upper 95% CI	0.181	0.155	0.106	0.308	0.285	0.217	0.509	0.384	0.461	0.370	0.394	0.703	0.626	0.620	0.643	0.514	—

Abbreviations: CTQ=Childhood Trauma Questionnaire, MD=Minimization/Denial, PN=Physical Neglect, EN=Emotional Neglect, SA=Sexual Abuse, PA=Physical Abuse, EA=Emotional Abuse, BIS=Barratt Impulsiveness Scale, T=Total, NP=Non-Planning, A=Attention, SC=Self-Control, M=Motor, WHODAS=World Health Organization Disability Assessment Schedule, P=Participation, A=Activities, GA=Getting Along, SC=Self-Care, M=Mobility, C=Cognition

Supplementary Figure S1. Heat map of Pearsons' correlation between trauma and impulsivity

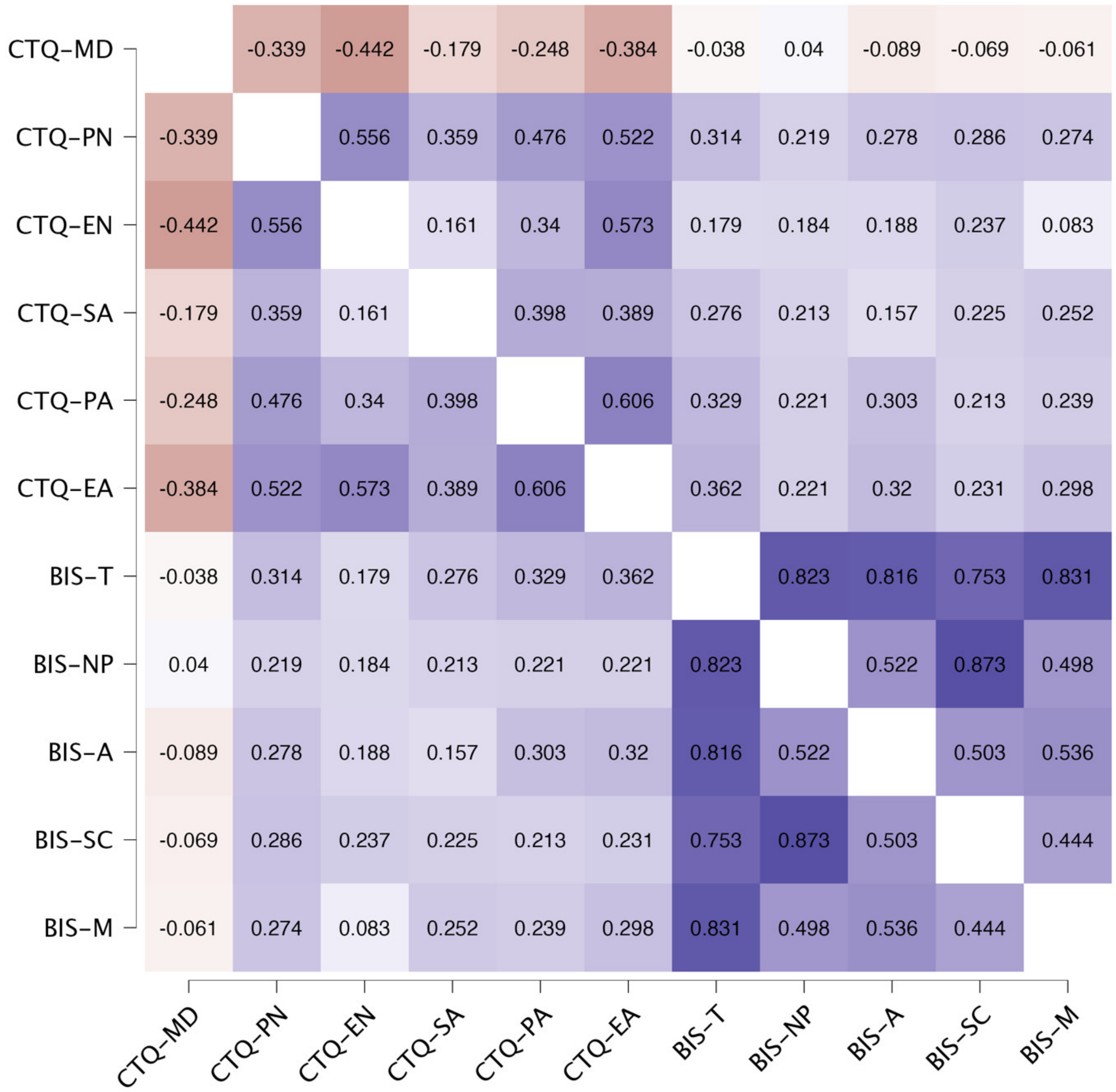

Supplementary Figure S2. Heat map of Pearsons' correlation between trauma and functionality

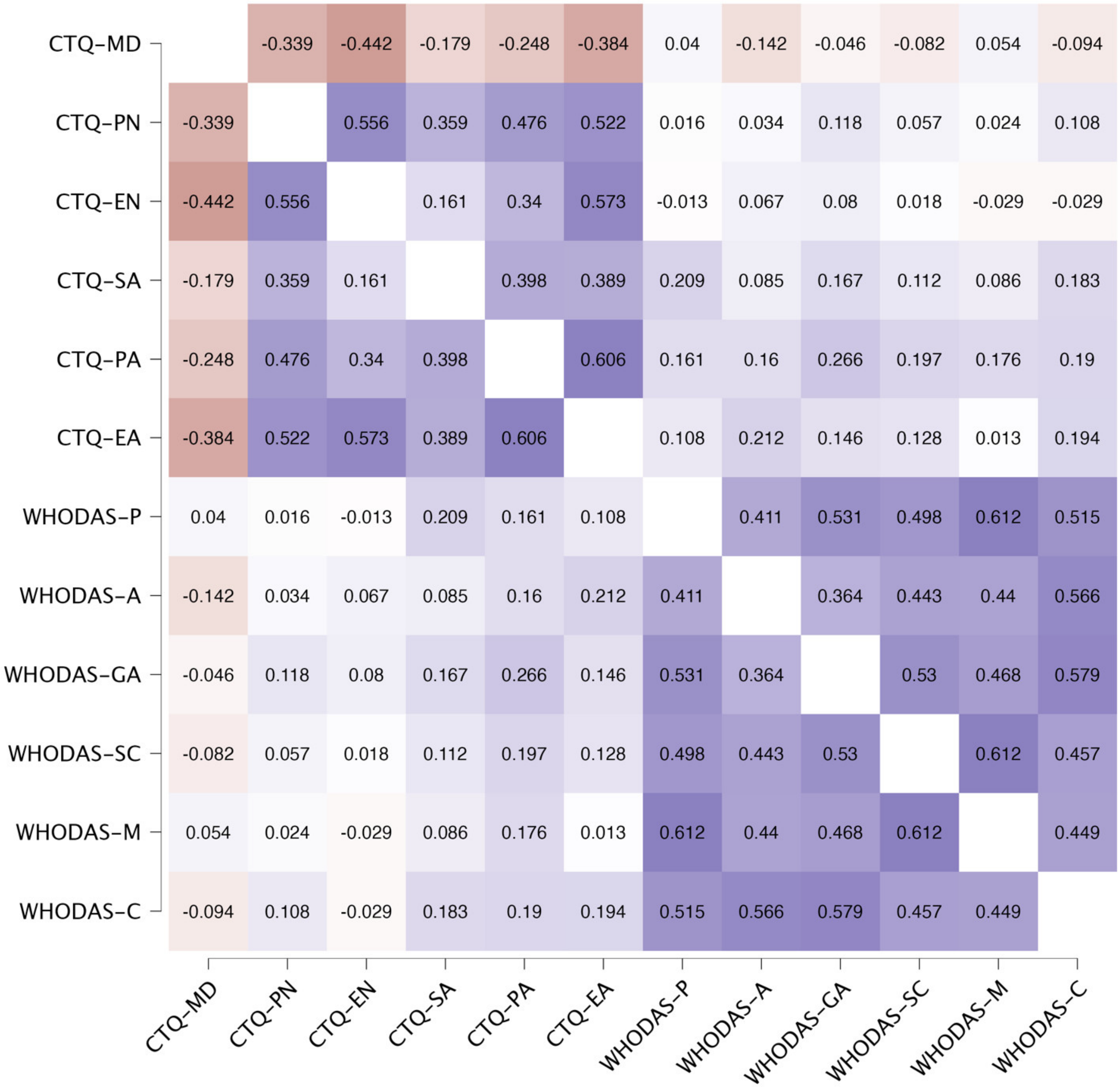

Supplementary Figure S3. Heat map of Pearsons' correlation between functionality and impulsivity

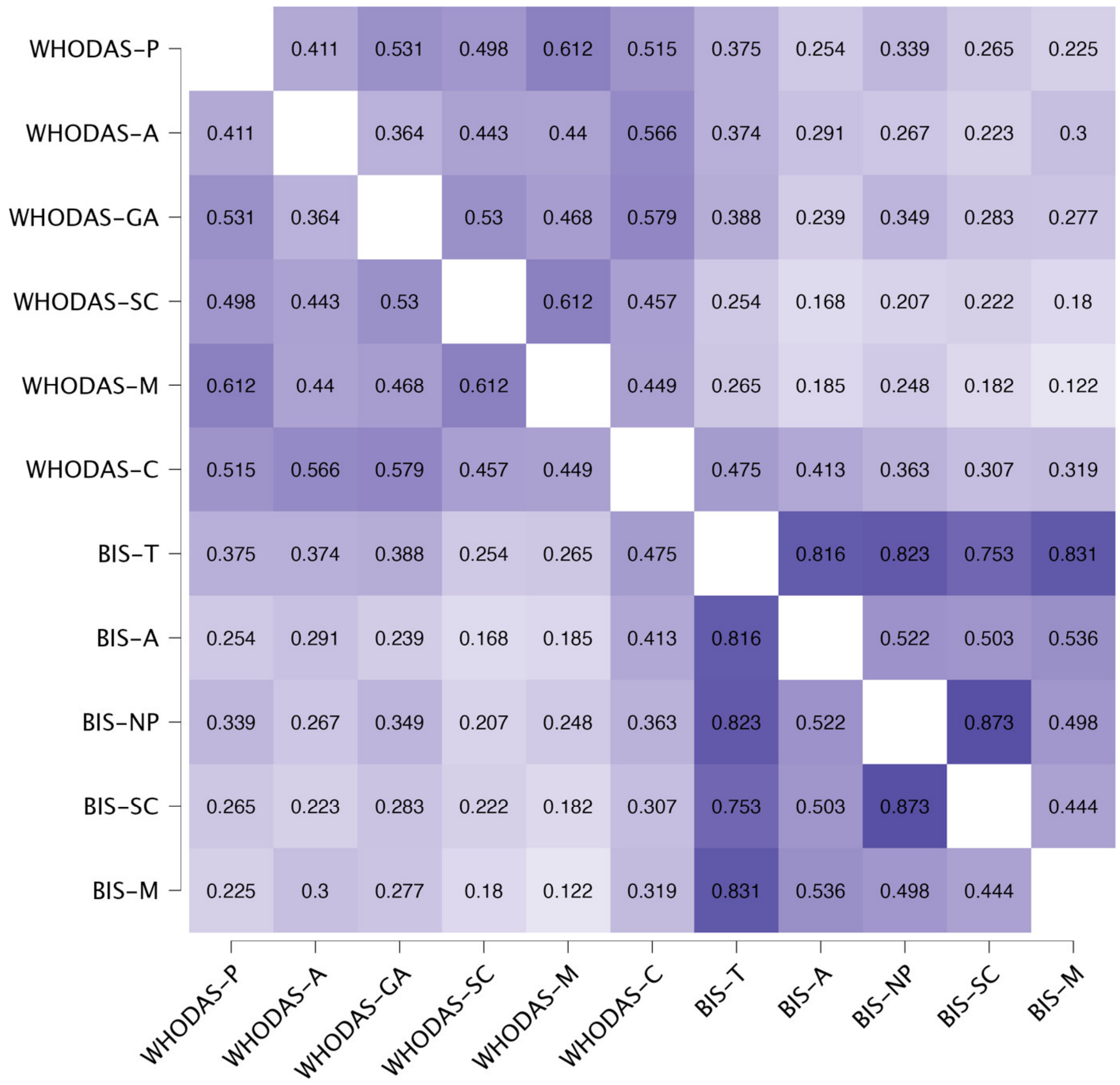

Supplement: Supplementary file 1 [file behavsci-15-01262-s001.zip › behavsci-3777586-supplementary.pdf]
